# Structural and Functional Diversity of Plant Virus 3′-Cap-Independent Translation Enhancers (3′-CITEs)

**DOI:** 10.3389/fpls.2017.02047

**Published:** 2017-11-29

**Authors:** Verónica Truniger, Manuel Miras, Miguel A. Aranda

**Affiliations:** Plant Virology, Centro de Edafología y Biología Aplicada del Segura, Consejo Superior de Investigaciones Científicas, Murcia, Spain

**Keywords:** plant virus, cap-independent translation, 3′-UTR, translation enhancer element, eukaryotic translation initiation factor, eIF4F, recombination, 5′–3′ RNA–RNA interaction

## Abstract

Most of the positive-strand RNA plant viruses lack the 5′-cap and/or the poly(A)-tail that act synergistically to stimulate canonical translation of cellular mRNAs. However, they have RNA elements in the 5′- or 3′-untranslated regions of their RNAs that are required for their cap-independent translation. Cap-independent translation enhancers (CITEs) have been identified in the genomic 3′-end of viruses belonging to the family *Tombusviridae* and the genus *Luteovirus*. Seven classes of 3′-CITEs have been described to date based on their different RNA structures. They generally control the efficient formation of the translation initiation complex by varying mechanisms. Some 3′-CITEs bind eukaryotic translation initiation factors, others ribosomal subunits, bridging these to the 5′-end by different mechanisms, often long-distance RNA–RNA interactions. As previously proposed and recently found in one case in nature, 3′-CITEs are functionally independent elements that are transferable through recombination between viral genomes, leading to potential advantages for virus multiplication. In this review, the knowledge on 3′-CITEs and their functioning is updated. We also suggest that there is local structural conservation in the regions interacting with eIF4E of 3′-CITEs belonging to different classes.

## Introduction

The m7G(5′)ppp(5′)N cap-structure that is covalently linked to the 5′ end and the poly(A) tail at the 3′ end are critical *cis*-acting elements during canonical eukaryotic mRNA translation, which is initiated with the recognition of the 5′-cap by the eukaryotic translation initiation factor (eIF) 4E and the poly(A) tail by the poly(A) binding protein (PABP). The scaffolding protein eIF4G binds to both and thus links the 5′- and 3′-ends of the mRNA resulting in its circularization. In plants, eIF4E and eIF4G shape the eIF4F complex, whereas eIF4A is probably loosely attached to the complex and does not co-purify (Hinnebusch and Lorsch, 2012). A peculiarity of plants is the existence of eIF(iso)4F, which includes the isoforms eIF(iso)4E and eIF(iso)4G. The significance of the existence of both complexes in plants is not fully understood; they seem to have overlapping functions but differential substrate preferences (Browning and Bailey-Serres, 2015) suggesting specialized roles.

Translation initiation is rate limiting and a highly regulated step (Aitken and Lorsch, 2012). While most plant-encoded mRNAs contain a 5′-cap and a poly(A)-tail that act synergistically to stimulate translation, ∼80% of known positive-strand RNA plant viruses lack one or both of these features in their genomic and subgenomic RNAs. Thus, non-canonical translation initiation occurs for most plant RNA viruses and functions independently of a 5′ cap or/and a poly(A) tail, using different *cis*-acting elements and mechanisms (Miras et al., 2017a). It is widely accepted that alternative mechanisms evolved by viruses to recruit the host’s translational machinery allow them to compete with the host mRNAs, avoiding host defenses at the level of translation.

Several viruses of the family *Tombusviridae* and the genus *Luteovirus* have been shown to contain cap-independent translation enhancers in the 3′-end of their genome(3′-CITEs) (Simon and Miller, 2013). Their mRNAs lack both the 5′-cap and 3′-poly(A) elements. In general, 3′-CITEs are able to recruit host translation initiation factors, which are subsequently brought to the opposite end of the RNA. Based on their different RNA structures, these 3′-CITEs can be classified into seven classes (Miras et al., 2017a). Their molecular mechanisms are also diverse, depending on different host translation initiation factors and varying circularization mechanisms. Some 3′-CITEs have been shown to interact with eIF4G, others with eIF4E and others directly with ribosome subunits. For some of the viral genomes carrying 3′-CITEs, the translation initiation complex formed at the 3′-end is brought to the opposite end by 5′–3′-RNA interactions based on sequence complementarity, in other cases by ribosome interactions with both ends.

3′-CITEs can remain functional when placed at the 5′-end, similar to the more abundant IRESes (internal ribosome entry sites), able to enhance cap-independent translation of many animal virus and some eukaryotic mRNAs (Balvay et al., 2009). But while IRESes are able to recruit the ribosome internally (independent of the 5′-*terminus*), for several 3′-CITEs it has been shown that the ribosome has to scan from the 5′-end of the RNA, as insertion of stable SL-structures at the 5′-*terminus* (Guo et al., 2001) or insertion of upstream out of frame AUGs abolished translation from the correct AUG (Rakotondrafara et al., 2006; Nicholson et al., 2010).

Construction and analysis of chimeric viruses have demonstrated that 3′-CITEs can be exchanged between viruses, suggesting that they are functionally independent RNA elements. Since the publication of the last review on 3′-CITEs (Simon and Miller, 2013), a new class of 3′-CITEs has been identified and the only known case of gain of a precise 3′-CITE in nature has been described (Miras et al., 2014). In this review, we update the knowledge on 3′-CITEs and their functioning.

## Classes of 3′-Cites Based on their Secondary Structures

To date, seven different classes of 3′-CITEs have been identified. Their structures are very variable, therefore 3′-CITE presence is difficult to predict and identification has to be based on functional analysis. In most cases activity has been assayed by flanking a reporter gene (for example luciferase) with viral 5′- and/or 3′-end sequences and analyzing the translation efficiency of these constructs preferably *in vivo* in protoplasts, since in some cases the 3′-CITEs are not functional *in vitro* in wheat germ extract (WGE).

### BTE

**FIGURE 1:**
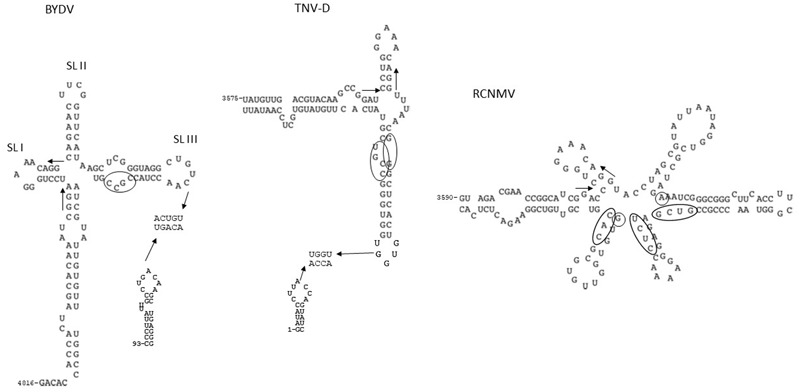
RNA secondary structures of BTEs, which are 3′-CITEs that interact with eIF4F through its eIF4G subunit. Assayed RNA secondary structures of representative BTEs of BYDV, TNV-D, and RCNMV, based on data derived from Wang et al. (2010). The conserved BTE-specific 17 nt are indicted with arrows (start/end). Bases protected by eIF4G, apart from the 17 nt stretch, are encircled (Kraft et al., 2013). Sequences proved or proposed to be involved in 5′–3′ interaction through sequence complementarity are shown for BYDV and TNV-D BTEs.

The 3′-CITE of *Barley yellow dwarf virus* (BYDV) is the best characterized and was named BYDV like-element (BTE) (**Figure 1**). It is present in all members of the genera *Luteovirus* (family *Luteoviridae*) and *Necrovirus, Dianthovirus*, and *Umbravirus* (family *Tombusviridae*) (Wang et al., 2010; Simon and Miller, 2013). The BTE structures consist of a long basal helix that connects with the rest of the viral genome, from which two (necrovirus), three (luteovirus) or five (dianthovirus) helices radiate, giving it a cloverleaf-like structure (Wang et al., 2010). BTEs contain a highly conserved sequence stretch of 17-nucleotides (nt), GGAUCCUGGgAaACAGG, where the underlined bases pair to form the stem of stem-loop I (SL-I). The bases in lower case can vary, but the consensus GNRNA (N is any base, R is a purine) in the apical loop is conserved (Koev et al., 2002). This sequence is supposed to form a particular structure that adds stability to the RNA hairpin, similar as shown for the GNRA-loop first identified in λ *boxB* RNA (Heus and Pardi, 1991; Legault et al., 1998). Wang et al. (1997) identified in the conserved 17 nt of the BTE of several viruses a sequence stretch that was complementary to the 18S rRNA and proposed a model where this complementarity results in direct binding of the ribosome to the BTE. Additionally, the unpaired bases at the hub of the helices and SL-II from BYDV BTE seemed to be critical, as mutations reduced its activity (Guo et al., 2000; Kraft et al., 2013).

### TED

**FIGURE 2:**
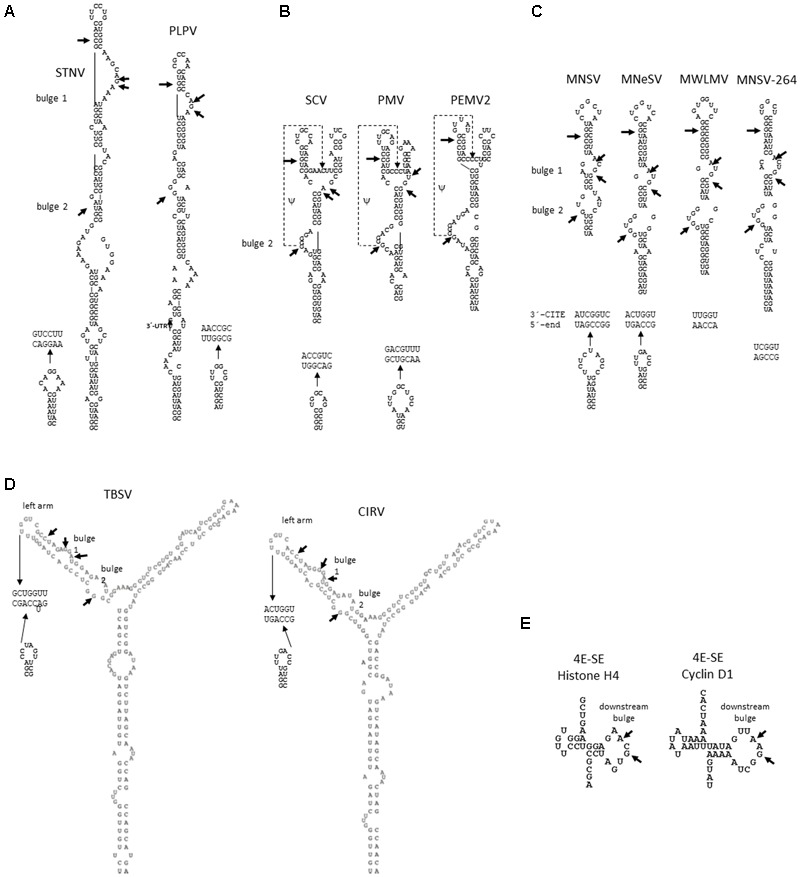
RNA secondary structures of TED, PTE, ISS, and YSS, which are 3′-CITEs shown or proposed to interact with eIF4F through its eIF4E subunit. **(A)** Assayed (PLPV) and predicted (STNV) TED structures, based on data derived from Blanco-Pérez et al. (2016). **(B)** Assayed PTE structures of SCV, based on data derived from Chattopadhyay et al. (2011) and of PEMV and predicted PTE structure for PMV, based on data derived from Wang et al. (2009). The pseudoknot (ψ) between C- and G-domain is indicated with a dashed line. **(C)** Assayed (MNSV and MNeSV) and predicted (MWLMV and MNSV-264) ISS (Nicholson et al., 2010; Miras et al., 2017b). **(D)** Predicted YSS of TBSV, as proposed by Fabian and White (2006) and CIRV, as proposed by Nicholson et al. (2013). **(E)** Assayed structures of eIF4E sensitivity elements (4E-SE) of Histone H4, based on data derived from Martin et al. (2011) and Cyclin D mRNAs, based on data derived from Culjkovic et al. (2006). Dashes point to conserved residues shown or predicted to be involved in eIF4E binding. The sequences proposed or shown to be involved in 5′–3′- interaction through sequence complementarity are shown. In the case of MNeSV the 5′–3′-interaction was proved with the 5′-UTR of CIRV.

The first 3′-CITE that was discovered was the one present in *Satellite tobacco necrosis virus* (STNV), a parasitic subviral RNA that encodes only its capsid protein; it was named translation enhancer domain (TED) (Danthinne et al., 1993; Timmer et al., 1993; Meulewaeter et al., 1998a). Its structure has been predicted to form a long stem-loop with several internal bulges and a final loop (van Lipzig et al., 2002; Blanco-Pérez et al., 2016), but this structure has not been confirmed experimentally (**Figure 2A**). The STNV TED was shown to be functional in enhancing translation *in vitro* and *in vivo*, being capable of functionally replacing a 5′-cap; also, it has been shown to confer cap-independent translation *in vitro* when moved to the 5′-untranslated region (UTR) of an uncapped reporter (Meulewaeter et al., 1998b). Recently, *Pelargonium line pattern virus* (PLPV), the recommended type member of the new *Pelarspovirus* genus belonging to the family *Tombusviridae*, was shown to contain a TED-like 3′-CITE (Blanco-Pérez et al., 2016). *In silico* and *in vitro* SHAPE analyses with the full-length PLPV genome together with structural conservation analysis supported that the TED element (**Figure 2A**) was considerably larger (49 nt) than previously proposed (Simon and Miller, 2013; Blanco-Pérez et al., 2016). An enlarged structure of the STNV TED was also suggested on the basis of *in silico* predictions with the complete STNV genome (Blanco-Pérez et al., 2016). Apart from STNV and PLPV, TED-like 3′-CITEs can be anticipated (although not yet experimentally verified) in the four members of the genus *Pelarspovirus, Pelargonium chlorotic ring pattern virus* (PCRPV), *Elderberry latent virus* (ELV), *Rosa rugosa latent virus* (RrLDV) and *Pelargonium ringspot virus* (PelRSV) (Blanco-Pérez et al., 2016) and additionally in *Calibrachoa mottle virus* (CbMV), a member of the genus *Carmovirus* (Simon and Miller, 2013). While *in vitro* translation experiments with the PLPV-TED in WGE were inconclusive (levels of newly synthesized p27 were similar in wild-type and truncated viral genomic transcripts, thus the 3′-CITE seemed not to be active) its translation enhancer activity could be confirmed for genomic (g) and subgenomic (sg) RNAs in *in vivo* translation experiments, showing that it is dependent on the presence of the genomic and subgenomic 5′-ends *in cis* (Blanco-Pérez et al., 2016).

### PTE

Batten et al. (2006) described a 3′-CITE in the 3′-UTR of *Panicum mosaic virus* (PMV) (*Panicovirus, Tombusviridae*) that was later shown to be similar in structure to the one identified in the umbravirus *Pea enation mosaic virus RNA 2* (PEMV2) (*Tombusviridae*) (Wang et al., 2009) (**Figure 2B**). These 3′-CITEs were named PTE (PMV or PEMV-like translational enhancer). PTEs are also found in several carmoviruses, as experimentally identified in *Saguaro cactus virus* (SCV) (Chattopadhyay et al., 2011) and proposed for *Pelargonium flower break virus* (PFBV), *Carnation mottle virus* (CarMV), *Honeysuckle ringspot virus* (HnRSV), *Pea stem necrosis virus* (PSNV), *Hibiscus chlorotic ringspot virus* (HCRSV) and *Galinsoga mosaic virus* (GaMV). The other two known panicoviruses, *Cocksfoot mild mosaic virus* (CMMV) and *Thin paspalum asymptomatic virus* (TPAV), as well as one of the known aureusviruses, *Pothos latent virus* (PoLV), also seem to contain a PTE (Simon and Miller, 2013). PTEs consist of two short helical branches connected by a C-rich or pyrimidine-rich, 3–6 nt bulge (C-domain) and downstream a guanylate-rich bulge in the stem (G -domain) (Wang et al., 2009). Wang et al. (2009) proposed the existence of a pseudoknot between the G-domain and the C-domain, but complementary mutations that were meant to reestablish disrupted base-pairing between these regions were unable to restore the PEMV PTE cap-independent translation activity lost through the mutations in only one domain, suggesting that maintenance of the sequence was also important (Wang et al., 2009).

### ISS

The shortest 3′-CITEs known have the shape of an “I” (I-shaped structure, ISS) and have been shown to be active in the 3′-UTRs of viruses belonging to different genera of the *Tombusviridae* family: the tombusvirus *Maize necrotic spot virus* (MNeSV) and the carmovirus *Melon necrotic spot virus* (MNSV) (Truniger et al., 2008; Nicholson et al., 2010; Miras et al., 2017b) (**Figure 2C**). The ISS consists of a stem-loop structure of about 60 nt with a 5–8 nt final loop and two internal bulges protruding from the stem. The first structure of the MNeSV determined by SHAPE analysis (Nicholson et al., 2010) was later further refined (Nicholson et al., 2013). Mutational analysis of the MNeSV showed that only a few mutations were tolerated, more specifically the ones conserving base-pairing in the stem regions (Nicholson et al., 2010). These authors proposed ISSs for the two 3′-CITEs that were previously identified in different MNSV isolates, MNSV-Mα5 and MNSV-264 (Truniger et al., 2008), and the proposed 3′-CITEs of three viruses belonging to the same family, the aureusviruses *Maize white line mosaic virus* (MWLMV) and *Johnsongrass chlorotic stripe mosaic virus* (JCSMV) and the tombusvirus *Cucumber Bulgarian virus* (CBV). Although there is considerable sequence divergence between the 3′-UTRs of all known MNSV isolates and MNSV-264, the 3′-CITE of MNSV-264 maintains the I-shape (Truniger et al., 2008). The structure of the MNSV-Mα5 ISS (Ma5TE), which is nearly invariant in the MNSV 3′-UTRs, has been solved by SHAPE analysis (Miras et al., 2017b); from the ISS structure of MNSV-Mα5 proposed by Nicholson et al. (2010) only the final SL (6 nt stem/7 nt loop) could be confirmed (Miras et al., 2017b). The Ma5TE, with its 45 nt, is the shortest ISS shown to be functional.

### YSS

Nearly all viruses belonging to the genus *Tombusvirus* (family *Tombusviridae*) can be predicted to have 3′-CITEs with a conserved Y-shaped structure (YSS) (Simon and Miller, 2013), as shown for the 3′-CITE of *Tomato bushy stunt virus* (TBSV) (Fabian and White, 2006) (**Figure 2D**). Another experimentally demonstrated YSS 3′-CITE is the one from *Carnation Italian ringspot virus* (CIRV, *Tombusvirus, Tombusviridae*) (Nicholson and White, 2008). The tombusviruses *Cymbidium ringspot virus* (CymRSV), *Cucumber necrosis virus* (CNV), *Artichoke mottled crinkle virus* (AMCV), *Pelargonium necrotic spot virus* (PNSV), *Grapevine Algerian latent virus* (GALV), and *Pelargonium leaf curl virus* (PLCV) have been proposed to contain a YSS (Simon and Miller, 2013). The three helices forming these YSSs are all substantially longer than the three helices found in the PTE (Fabian and White, 2006). Mutations in the YSS of TBSV which altered the structure, thereby affecting stem or bulge formations, affected translation mediated by this 3′-CITE, while those that maintained the structure of the three stems had no substantial effect on translation (Fabian and White, 2006), supporting the importance of the YSS for its activity.

### TSS

**FIGURE 3:**
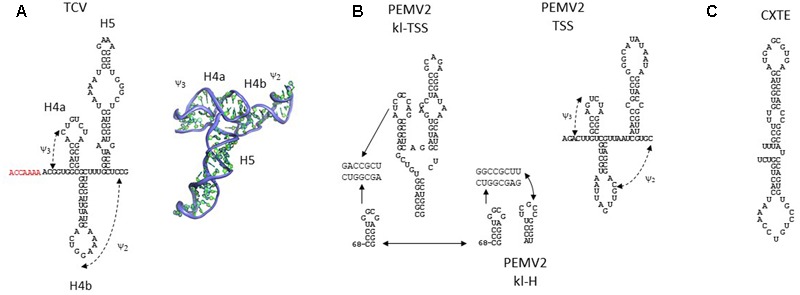
RNA secondary structures of T-shaped 3′-CITEs and CXTE. **(A)** Assayed two- (McCormack et al., 2008) and three-dimensional structure Zuo et al. (2010) of the TCV TSS, based on data derived from McCormack and Zuo and collegues, respectively. Pseudoknots (ψ_2_ and ψ_3_) are indicated with a dashed line. Hairpins are H4a/b and H5. Additional nucleotides present in the extended TSS model appear in red (Le et al., 2017). **(B)** Assayed RNA secondary structures of the two PEMV2 TSS, kl-TSS (Gao et al., 2012, 2013) and TSS (Gao et al., 2014), based on data derived from Gao and colleagues. 5′-end sequence interacting by sequence complementarity with the kl-TSS and with kl-H is shown. **(C)** Assayed RNA secondary structure of CXTE, the newest 3′-CITE, based on our data derived from Miras et al. (2014).

This 3′-proximal translation enhancer, first discovered in *Turnip crinkle virus* (TCV, *Carmovirus, Tombusviridae*) (McCormack et al., 2008) has a three-dimensional T-shaped structure (TSS), as predicted by molecular modeling and confirmed by small-angle X-ray scattering (SAXS)/NMR (Zuo et al., 2010). This is the first and only resolved 3D structure of a 3′-CITE. The TSS contains three hairpins and two pseudoknots that fold into a structure similar to tRNAs (Zuo et al., 2010) (**Figure 3A**). A similar structure was proposed for the related carmovirus *Cardamine chlorotic fleck virus* (CCFV). Additionally, apart from its PTE two functional TSSs have been identified in the PEMV2-3′-UTR, one upstream of the PTE, the kl-TSS, and the other near the 3′-end of the genomic RNA, the 3′-TSS, with both predicted to fold into structures similar to tRNAs (Gao et al., 2012, 2013) (**Figure 3B**). Although both are essential for virus accumulation *in vivo*, mutations that disrupted the TSS near to the 3′-end had no effect on translation (Gao et al., 2013). More recently, the same authors have shown that the 3′-TSS is also functional in translation *in vitro* when deleting the kl-TSS and the PTE (Gao et al., 2014).

Unlike the tRNA-like structures present in tobamo-, tymo-, and rubiviruses, which are aminoacylated and contribute to translation together with the 5′-cap, TCV and CCFV genomic RNAs are uncapped and their TSS are present in an internal location, thus they cannot be aminoacylated (Dreher and Miller, 2006; Dreher, 2009).

The RNA-dependent RNA polymerase (RdRp) has been shown to bind the 3′-end of the TCV genome leading to a conformational change of the TSS region (Yuan et al., 2009). The resulting conformation is suggested to be thermodynamically favored, as the original TSS conformation is formed again by experimentally degrading the RdRp. This RdRp-mediated conformational switch is expected to prevent TSS function, allowing replication to initiate at the 3′-end (Yuan et al., 2010). Thus, it has been proposed that in TCV an integrative network of interactions is required for virus accumulation, with different SLs involved in regulation of TCV translation and replication (Yuan et al., 2012). Very recently a new structural model for an extended TSS has been proposed that includes the A-rich region upstream adjacent to the TSS (in red in **Figure 3A**). It was shown that this region is implicated in RdRp binding, inducing the conformational change of the TSS possibly by disruption of the H4a/ψ3 pseudoknot that leads to loss of the H4b/H5 interaction (Le et al., 2017). This conformational switch is expected to interrupt translation through disruption of the TSS while promoting replication.

### CXTE

The newest 3′-CITE, CXTE (CABYV-Xinjiang-like translation element), is also small (55 nt) and consists of two helices protruding from a central hub (Miras et al., 2014). Since it is different from the ones identified until now it belongs to a new class of 3′-CITEs. It has been identified in the new MNSV-N isolate as a second 3′-CITE apart from its ISS Ma5TE (**Figure 3C**). This translational enhancer was named CXTE, as it had been acquired by MNSV from an Asiatic isolate of *Cucurbit aphid-borne yellows virus* (CABYV Xinjiang, genus *Polerovirus*, family *Luteoviridae*) (Miras et al., 2014). Thus, MNSV-N has two 3′-CITEs, the typical MNSV ISS and the CXTE.

It can be postulated that more types of 3′-CITEs may exist, as the predicted secondary structures of the 3′- RNA ends of several viruses of the family *Tombusviridae* do not resemble any of the known 3′-CITE classes (Simon and Miller, 2013). Thus, although the final goal of 3′-CITEs in cap-independent translation seems to be common, their sequences and structures are highly variable.

## Host Eukaryotic Translation Initiation Factors Involved in Different Viral 3′-Cite Activities

### BTE

The BTE has been shown to bind eIF4F through the eIF4G subunit of the heterodimer (Treder et al., 2008). In WGE depleted of cap-interacting factors addition of the isoform eIFiso4F also allowed BYDV BTE-mediated translation to recover, but to a lesser extent than addition of eIF4F. Interestingly, lower levels of eIF4F/eIFiso4F were required for efficient BTE-mediated translation than for cap-dependent translation (Treder et al., 2008). Binding of eIF4G to the BTE occurs with high affinity (*K*_d_ = 177 nM) and is sufficient to facilitate translation alone. On the other hand, eIF4E alone does not promote BTE-mediated translation, but the presence of both subunits of eIF4F results in a 25% increase in translation efficiency, while the affinity of BTE binding to eIF4F is four-fivefold higher (*K*_d_ = 37 nM) (Treder et al., 2008). This could be due to a conformational change in the eIF4G subunit induced by eIF4E (Zhao et al., 2017). Through footprinting experiments with the BYDV, TNV-D and RCNMV BTEs it was shown that eIF4G protects the SL formed by the 17-nt conserved sequence and several additional residues as marked in **Figure 1** (Kraft et al., 2013). Analysis of the minimal eIF4G fragment required for BYDV BTE binding and activity revealed that it had to include the eIF4A and eIF3 sites and an adjacent RNA-binding domain, but the eIF4E and PABP binding sites were not necessary (Kraft et al., 2013; Zhao et al., 2017). Sharma et al. (2015) proposed that after eIF4F binds to the BTE also eIF4A, eIF4B, and ATP bind, forming the helicase complex and recruiting the 40S ribosomal subunit. Although the 40S ribosomal subunit can bind the BTE in the absence of the helicase complex, its presence improves binding affinity. eIF4A, eIF4B, and ATP are also able to enhance binding of eIF4G to BTE of BYDV in the absence of the ribosome (Zhao et al., 2017). The authors proposed that the binding of eIF4A/eIF4G/ATP alters the BTE structure resulting in enhancement of the binding affinity between BTE and eIF4G followed by recruitment of the 40S ribosome subunit directly to the BTE. However, in a previous model it was proposed that base-pairing between a sequence present in the conserved 17 nt of the BTE that was complementary to the 18S rRNA could result in direct ribosome binding to the BTE (Wang et al., 1997). The presence of these complementary sequences partially in internally base-paired stretches could explain the requirement of helicase activity for increased ribosome binding to the BTE and thus for BTE-mediated translation (Sharma et al., 2015).

### TED

The 3′-CITE of STNV, TED, has been shown to bind wheat eIF4F and eIFiso4F, but with preference for the first complex, with a *K*_d_ of 30 and 50 nM, respectively (Gazo et al., 2004). This interaction is required for TED activity, as in WGE depleted in cap-associated eIFs, TED-mediated translation was strongly reduced, but could be restored by the addition of eIF4F or its isoform. The binding to the individual eIF4F subunits was studied in competition assays. While the eIF4G binding to TED was not affected by the addition of unlabeled TED *in trans*, eIF4E binding was specifically inhibited (Gazo et al., 2004). Thus, although binding affinity of TED to eIF4E or eIFiso4E alone was found to be more than 10 times lower than to the respective eIF4F or eIFiso4F complexes, complex binding likely occurred through interaction with the eIF4E subunit. Mutation of one of the two highly conserved tryptophans of the cap-binding pocket of eIF4E strongly reduced its binding affinity to STNV TED (Wang et al., 2009), suggesting that this 3′-CITE requires an intact cap-binding pocket as shown for PTE and ISS (see below).

### PTE

In contrast to the previous 3′-CITEs, the PTE of PEMV2 has been shown to bind eIF4E with high affinity (*K*_d_ = 58 nM) even in the absence of eIF4G, with the binding affinity to eIF4F being very similar (Wang et al., 2009). Mutational analysis of eIF4E allowed concluding that eIF4E binding to PTE of PEMV2 correlated with its cap-independent translation activity (Wang et al., 2009). In spite of the strong binding of TED to eIF4E, this subunit alone was not able to enhance TED-mediated translation (Wang et al., 2009). Thus, this 3′-CITE also requires the eIF4F complex for activity. Footprinting experiments with eIF4E showed protection in the region around the pseudoknot of the PEMV2 PTE (formed by G- and C-domains) (Wang et al., 2011). In the lateral bulge containing the G-domain, one highly reactive G-residue (G17) has been proposed to protrude into the cap-binding pocket, being clamped by eIF4E (Wang et al., 2011) (**Figure 2B**).

As shown for STNV TED, mutation of both highly conserved tryptophans located in the cap-binding pocket of eIF4E was shown to strongly reduce its binding to PEMV2 PTE (Wang et al., 2009), suggesting that also for this 3′-CITE an intact cap-binding pocket is required. The much higher eIF4E binding affinity of PTEs may be achieved by binding the pseudoknot, which is absent in the ISS 3′-CITEs.

### ISS

The ISS of MNeSV has been shown to interact with wheat eIF4F in WGE (Nicholson et al., 2010). In this case, eIF4F binding occurred through its eIF4E subunit, but additions of purified recombinant eIF4E or eIF4G alone were not able to restore translation in WGE depleted in cap-associated eIFs. The *K*_d_ of binding of eIF4F to the MNeSV ISS was estimated to be around 190 nM. Interestingly, two G residues, shown to be highly flexible in the structure, were found to be important for eIF4F binding, as deletion (G13) or mutation (G47C) strongly reduced its eIF4F binding capacity (Nicholson et al., 2010). These G residues can also be identified in bulges in the structures of the other ISSs (assayed or predicted) (**Figure 2C**).

In the case of the Ma5TE ISS, genetic and biochemical evidence for an interaction between Ma5TE and eIF4E exists (Nieto et al., 2006; Miras et al., 2017b). One amino acid change in melon eIF4E causes loss of susceptibility to MNSV due to the strong reduction in the cap-independent translation efficiency mediated by its Ma5TE (Nieto et al., 2006; Truniger et al., 2008). In agreement with the results obtained for MNeSV ISS, *in vitro* binding assays showed that Ma5TE also binds eIF4F: eIF4F_p20_, formed by purified recombinant melon eIF4E with a 20 kDa truncated eIF4G fragment, was shown to be competent for Ma5TE binding, with the *K*_d_ estimated to be 2 μM (Miras et al., 2017b). The eIF4E residues that are important for Ma5TE translation activity were studied by mutational analysis: apart from the residue responsible for the loss of susceptibility, cap-binding pocket residue tryptophan 82 and residues of eIF4E involved in its interaction with eIF4G through its canonical and non-canonical binding domains were also shown to be required for *in vivo* Ma5TE activity (Miras et al., 2017b,c). Thus, although binding of eIF4F to Ma5TE seems to occur through eIF4E, the eIF4F complex is also required for efficient cap-independent translation driven by Ma5TE. Again, as previously shown for STNV TED and PEMV2 PTE (Wang et al., 2009), an intact cap-binding pocket seems to be important for MNSV ISS activity (Miras et al., 2017b).

Mapping of the eIF4F binding sites in Ma5TE by footprinting analysis revealed that, in the presence of eIF4F_p20_, the highly accessible residue A32 was protected, whereas G17 and C28 (**Figure 2C**), which were paired and thus not accessible without eIF4F, became accessible. Mutations in these residues affected the *in vivo* cap-independent translation activity of Ma5TE, while no binding of eIF4F_p20_ to Ma5TE A4109C could be observed *in vitro* (Miras et al., 2017b). These three residues (adenosine and G/C pair) can be identified in similar positions in the other ISSs (**Figure 2C**). Interestingly, mutation of the corresponding adenosine residue in MNeSV 3′-CITE (reducing its flexibility by pairing it) also strongly affected the activity of this ISS (Nicholson et al., 2010). This suggests that this adenosine could be involved in eIF4F binding in all ISS 3′-CITEs. Similarly, the two unpaired guanosines shown to be involved in MNeSV ISS binding to eIF4F are conserved in all other ISSs, suggesting that these residues could be also involved in eIF4F binding through eIF4E (arrows in **Figure 2C**).

Interestingly, the ISS of MNSV-264 also conserves these residues (**Figure 2C**), although it has been shown to be able to function in the absence of eIF4E. MNSV-264 is able to infect melon plants with reduced eIF4E expression due to its specific silencing, in contrast to MNSV-Mα5 that is unable to infect (Rodríguez-Hernández et al., 2012). Thus, it can be hypothesized that MNSV-264 ISS may be active in cap-independent translation with eIF4E but additionally is also able to function in its absence using eIFiso4E or without the 4E factor. Multiplication of several potyviruses has been shown to be functional with both eIF4E and eIFiso4E (Ruffel et al., 2006; Hwang et al., 2009; Jenner et al., 2010).

### YSS

Another 3′-CITE that is functional with either eIF4F or eIFiso4F is the YSS of CIRV. The strongly reduced YSS-mediated translation activity in factor-depleted WGE could be restored by addition of eIF4F or eIFiso4F (Nicholson et al., 2013). In an RNA-affinity chromatography experiment, the CIRV YSS complexed with all four eIF4F components and the same result was obtained for the TBSV YSS (Nicholson et al., 2013), suggesting that this mechanism is general for these types of 3′-CITEs.

### CXTE

The eIFs required for activity of the newly identified third MNSV-3′-CITE (CXTE), with high homology to the 5′-end of the CABYV 3′-UTR, are still unknown. The CXTE is functional in melon plants silenced for eIF4E, thus it may be eIF4E-independent (Miras et al., 2014).

### TSS

In contrast to the other 3′-CITEs described above, no eIFs that bind to the TCV TSS have been described to date, although translation of reporter constructs flanked by TCV 5′- and 3′-UTRs has been shown to be reduced in the absence of eIF4G in an *Arabidopsis* eIF4G-deficient mutant (Yoshii et al., 1998, 2004). This structure has been shown to directly recruit and bind to the P-site of the 60S subunit (Stupina et al., 2008) and the 80S ribosome (Zuo et al., 2010). In the PEMV2 3′-UTR the two TSSs identified have been both shown to bind to the 60S subunit and the 80S ribosome, but one of them, the kl-TSS, additionally binds to the 40S ribosomal subunit (Gao et al., 2013, 2014). In PEMV2, all three 3′-CITEs, the PTE and the two TSSs, are required for efficient translation (Gao et al., 2014).

## Mechanisms for Bringing the eIfs Bound to the 3′-Cite to the 5′-End

As translation occurs in the 5′–3′ direction of the mRNA, the eIFs bound to the viral 3′-CITEs have to be brought to the 5′-end for translation initiation complex formation. In most viral RNAs with 3′-CITE mediated cap-independent translation, this has been proposed or shown to be achieved by a kissing-loop interaction based on sequence complementarity between the 3′-CITE and the 5′-end (most often present in the 5′-UTR, but in some cases in the beginning of the first ORF). In these cases, the 3′-CITE activity is 5′-end dependent and mutations disrupting base-pairing affect translation, while compensatory mutations restore activity. The interacting sequences are usually unpaired, thus located in conserved apical loops of hairpins (Simon and Miller, 2013).

### BTE

One example is the BTE-mediated translation of BYDV: the sequence in the loop of SL-III from the BTE of BYDV is complementary to the loop of SL-D in the 5′-UTR. This complementarity is required for efficient translation mediated by BYDV BTE (Guo et al., 2001; Wang et al., 2010) (**Figure 1**). This BTE was functional even if complementarity was achieved by insertion of foreign sequences outside the BTE (from viral or non-viral origin) (Rakotondrafara et al., 2006). The presence of eIF4A, eIF4B and ATP was shown to enhance binding of eIF4G to BTE, possibly due to BTE structure alteration (Zhao et al., 2017). Two alternative models for cap-independent translation by BTE have been proposed (Miras et al., 2017a): either after binding of eIF4F, eIF4A, and eIF4B to BTE the 40S ribosomal subunit is directly recruited to it through sequence complementarity between the BTE and the 18S rRNA and delivered to the 5′-end through the 5′–3′ complementary interaction; or after binding of eIF4A and eIF4B to BTE followed by ATP hydrolysis, its affinity for eIF4F is increased but first the 5′–3′ interaction has to bring the formed complex close to the 5′-end before it recruits the 43S preinitiation complex. In both cases ribosome scanning occurs from the 5′-end. The complementarity between BTE and 5′-UTR is maintained among BTEs, suggesting that this interaction may be required for translation enhancement activity by BTEs.

The only exception is the BTE-like 3′-CITE (named 3′TE-DR1) of *Red clover necrotic mosaic virus* (RCNMV; genus *Dianthovirus*, family *Tombusviridae*) (**Figure 1**), where no effect on translation was observed by mutating complementary sequences between BTE and 5′-UTR (Sarawaneeyaruk et al., 2009). In this case, in addition to its BTE, an A-rich sequence (ARS) (60 nt upstream to it) with strong affinity for PABP was identified in the RCNMV 3′-UTR and both ARS and 3′-CITE, were shown to coordinately recruit eIF4F or eIFiso4F and the 40S ribosomal subunit to the viral RNA (Iwakawa et al., 2012). The ARS is conserved in all dianthoviruses. Interestingly, while cap-independent translation of the RNA1 of this bipartite virus is controlled by a BTE that depends on eIF4F for activity, no 3′-CITE could be identified in its RNA2. Translation of this RNA2 has been shown to depend on eIFiso4F, suggesting that the eIFs required for cap-independent translation of the RNAs of this bipartite virus differ (Tajima et al., 2017).

### TED

Again, the TED-mediated translation of STNV is dependent on the presence of the 5′-end *in cis* (Meulewaeter et al., 1998a). The eIF4F or its isoform bound to TED was predicted to reach the ATG through a RNA interaction with the apical loop of the 5′ end based on sequence complementarity (**Figure 2A**). However, this could not be confirmed by mutational analysis, as disruption of this potential base-pairing reduced translation only slightly, while restoring complementarity by mutations at the opposite end did not restore translation (Meulewaeter et al., 1998a). Thus, the authors suggested that base-complementarity was biologically relevant but not crucial for the constructs and assays used. But for the recently described TED of PLPV, a long distance interaction between TED and 5′-end based on sequence complementarity has been shown to occur (**Figure 2A**). The complementary sequence in the 5′- end (involving 6–8 bases) is located in the viral p27 ORF (Blanco-Pérez et al., 2016), possibly because its 5′-UTR is very short. This 5′–3′ interaction is required for efficient translation of the viral genome and thus for infectivity. Complementary sequence stretches are conserved in other viral genomes proposed to contain TED-like 3′-CITEs, suggesting that 5′–3′ RNA-RNA interactions are necessary in all TED-mediated translation mechanisms to bring the eIF4F bound to the 3′-UTR to the opposite end (Blanco-Pérez et al., 2016). In line with this, moving of the TED of STNV to the 5′-end allowed TED-mediated translation to occur in the absence of circularization (Meulewaeter et al., 1998b).

### PTE

All viral genomes where PTEs have been identified, excluding the PEMV2, contain sequence stretches in their 5′-ends that are complementary to a loop in its structure (Simon and Miller, 2013) (**Figure 2B**). In the case of the carmovirus SCV the functionally important 5′ complementary sequence is located in the loop of a hairpin in the coding region of p26 (Chattopadhyay et al., 2011), possibly because of its short 5′-UTR, while in the panicovirus PMV the complementarity is with the first hairpin of the 5′-UTR (Batten et al., 2006). For this PTE it was shown that maintenance of its distance to the 5′-UTR is important for its efficient activity (Chattopadhyay et al., 2014). For the other virus genomes with proposed PTEs, complementary sequences between the PTE (Nicholson et al., 2010) and the 5′-end have been identified (Simon and Miller, 2013), suggesting that the mechanism of circularization is common. As will be described below, the PEMV2 PTE depends on another 5′–3′ interaction, the kl-TSS that helps to bring the eIF4F complex to the 5′-end.

### ISS

As with the other 3′-CITEs described above, 5′–3′-UTR interactions based on sequence complementarity have been identified between the apical loop of the only SL forming the ISS of MNeSV and the loop of the first SL of the 5′-UTR (Scheets and Redinbaugh, 2006) and shown to be required for virus genome translation and multiplication (Nicholson et al., 2010) (**Figure 2C**). This interaction was shown not to interfere with eIF4F binding, while ribosome scanning was dependent on the ISS and occurred from the 5′-end (Nicholson et al., 2010). Similarly, ISS-mediated translation of MNSV was shown to be 5′-UTR dependent (Truniger et al., 2008) and complementarity between bases in the loops of the ISS and SL1 of the 5′-UTR exists (**Figure 2C**). For the two aureusviruses MWLMV and JCSMV and the tombusvirus CBV as well, complementary sequence stretches between the 5′-end and the loop of their predicted ISSs could be identified. Thus, in the ISS-mediated translation activity, the eIF4F bound to the 3′-CITE reaches the 5′-end through 5′–3′ interaction.

### YSS

The YSS-mediated translation efficiency of TBSV has also been shown to depend on a 5′–3′-UTR interaction between its YSS and 5′-UTR. Mutational analysis that disrupts and restores complementarity confirmed this interaction, which is required for protein synthesis and virus multiplication (Fabian and White, 2004, 2006) (**Figure 2D**). Moreover, the YSS of CIRV was also shown to require a 5′–3′ interaction between its YSS and 5′-UTR, based on sequence complementarity, in order to bring the eIF4F (or isoform) complex bound to the YSS to the 5′-end (Nicholson and White, 2008; Nicholson et al., 2013), suggesting that 5′–3′ interactions are common in YSS-mediated translations (**Figure 2D**). Additionally, for both TBSV and CIRV YSS ribosome scanning has been shown to occur from the 5′-end (Fabian and White, 2006; Nicholson et al., 2013).

### CXTE

In MNSV-N, the CXTE activity has also been shown to depend on the presence of the 5′-UTR of MNSV *in cis* (Miras et al., 2014). But in this case, although complementary sequence stretches between the CXTE and the 5′-UTR exist, mutational analysis did not support its importance in CXTE-mediated translation. In the 3′-UTR of MNSV-N two 3′-CITEs, CXTE, and ISS Ma5TE, are present. In reporter constructs having only the CXTE sequence (not the entire 3′-UTR) flanking the luciferase gene at the 3′-end, its activity in translation was shown to depend also on the presence of the 5′-UTR *in cis* (Miras et al., 2014). This result excludes the possibility that the CXTE takes advantage of the possible 5′–3′ interaction occurring between Ma5TE and 5′-UTR for its activity. Thus, the mechanism of the CXTE in cap-independent translation is currently unknown.

### TSS

In the case of TCV, no base-pairing between its TSS 3′-CITE and 5′-UTR could be identified. Here, the 60S ribosomal subunit bound to its TSS is expected to reach opposite end through a protein bridge by binding to the 40S subunit bound to its 5′-UTR (Stupina et al., 2011). Thus, the 80S ribosome would simultaneously bind to both TCV UTRs and this would explain why ribosomal binding to the 5′-UTR was altered in the presence of the 3′-UTR.

As in the case of PEMV2 the PTE is not able to contact the 5′-end by direct sequence complementarity, the eIF4F complex bound to this 3′-CITE is brought to the 5′-end by a kissing-loop interaction occurring through the adjacent TSS-like 3′-CITE (kl-TSS) which binds the 60S ribosomal subunit (Gao et al., 2012, 2013) (**Figure 3B**). The hairpin at the 5′-end involved in this interaction is located in the coding region of the first ORF. This interaction was shown to be critical for virus translation and multiplication, and its loss affected translation to a greater extent than deletion of the adjacent PTE. This suggested that the 5′–3′ interaction supported additional translation functions besides bringing the PTE bound eIF4E to the 5′-end (Gao et al., 2012). The kl-TSS is able to simultaneously bind to the ribosome and the 5′-end (Gao et al., 2013). Additionally, the second TSS-like 3′-CITE in the 3′-UTR of PEMV2 has been shown to require a small hairpin upstream of the TSS named kl-H with an apical loop that is able to interact by sequence complementarity with the same 5′-proximal hairpin that is utilized by kl-TSS (Gao et al., 2014). Recently, it has been shown that while efficient translation of the gRNA requires only two of the three PEMV2 3′-CITEs, the kl-TSS and the PTE (Du et al., 2017), efficient translation of the sgRNA additionally requires the second ribosome binding TSS. Here the RNA–RNA interaction of the 5′-end of the sgRNA with the kl-TSS is required (Gao and Simon, 2017). The requirement for different translation mechanisms could be a way for regulating virus protein expression at the different stages of the viral infectious cycle. In conclusion, all three PEMV2 3′-CITEs are required for efficient virus translation.

## Are Residues Involved in eIf4E Binding Conserved in 3′-Cites?

Several residues of these 3′-CITEs have been described to be protected in the presence of eIF4E and to be important for 3′-CITE activity: in the case of the PEMV2 PTE the G-domain residues, especially the first one (G17 in **Figure 2B**) (Wang et al., 2011); in the case of the MNeSV ISS two G residues were protected: the first (G13) downstream of the apical loop in the second lateral bulge 2, resembling this PTE G-domain and the second (G47), present upstream of the apical loop in the first bulge 1 (**Figure 2C**) (Nicholson et al., 2010); and finally an unpaired adenosine adjacent to this second G residue (A32) that was protected in MNSV ISS as well as the G/C pair (G16/C28) in the stem of the final stem-loop (**Figure 2C**) (Miras et al., 2017b). Interestingly, in the Cyclin D1 and Histone H4 mRNAs the eIF4E-sensitivity elements (4E-SE) shown to be involved in eIF4E binding without the need of cap (Culjkovic et al., 2006; Martin et al., 2011) also have A and G residues in the downstream loop of its double SL structure (**Figure 2E**), similar to the ISS. Toe-print analysis of the H4 4E-SE in the presence of eIF4E showed RT-stops at the A and the following C, suggesting that eIF4E binding involves this loop (Martin et al., 2011).

**FIGURE 4:**
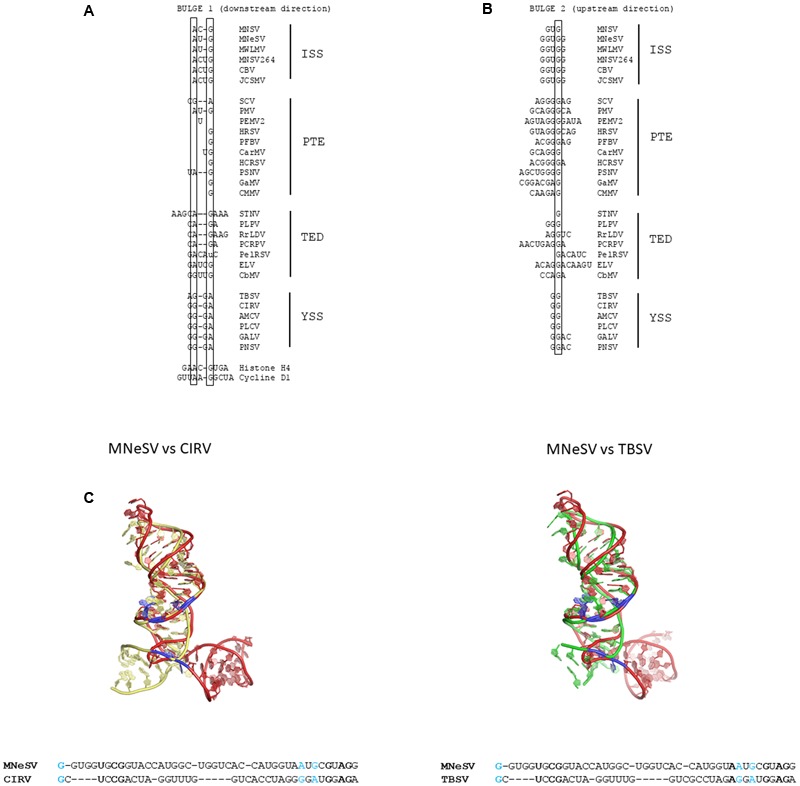
Conservation of unpaired 3′-CITE residues suggested to be involved in eIF4E binding. **(A)** Residues of the first bulge present downstream of the final stem-loop of ISS, PTE, TED, and YSS CITEs and of the histone H4 and cyclin D1 4E-sensitivity elements. **(B)** Residues of the second bulge present upstream of the final stem-loop of these 3′-CITEs. Conserved adenosine [in **(A)**, similar to A32 of MNSV ISS] and guanosine residues [similar in **(A)** to G47 of MNeSV ISS and in **(B)** to G13 of MNeSV ISS and the highly reactive G17 residue from the G-domain of PTE] are framed. **(C)** 3D structural alignment [Rclick server (Nguyen et al., 2017)] of the left arm of the YSS of CIRV and TBSV (colored in yellow and green, respectively) with the ISS of MNeSV (red colored), based on the predicted tertiary structures [RNAComposer server (Popenda et al., 2012)]. YSS of CIRV and TBSV and ISS of MNeSV are based on data derived from Nicholson et al. (2010, 2013), respectively. Below, alignment of sequence stretch of both structures aligning in the 3D prediction (Rclick server). Identity of residues is emphasized in bold. Adenosine and guanosine residues predicted to be important for eIF4E binding are shown in blue in the 3D structural alignment and in red in the sequence alignment.

Analysis of the residues present downstream the apical loop in bulge 1 of the assayed or predicted structures of TED 3′-CITEs allows for the identification, as in all the ISSs, of A and G residues (adjacent or separated by a maximum of 2 nt) conserved in all of them [**Figures 2A,C** (arrows), **4A**]. Thus, these two residues could be involved in eIF4E binding in these 3′-CITEs. In the case of the PTE structures, these two residues are less conserved (**Figures 2B, 4A**). Additionally, one or two guanosine residues can also be identified in ISS and TED CITEs upstream of the apical loop in bulge 2 in a similar position as the G-domain of PTEs, suggesting their possible role in eIF4E binding (**Figures 2A–C, 4B**). Also, the paired G/C present in the final stem that became accessible in the presence of eIF4E in MNSV ISS can be identified in all TED and PTE CITEs as a G/C (or C/G) pair (**Figures 2A–C**).

The five residues proposed above to play a role in eIF4F binding through eIF4E can also be identified in the YSSs, located in the left arm of the Y (**Figures 2D, 4A,B**). If 3D structural predictions of the left helix of the YSS of CIRV or TBSV are superimposed on the prediction of the ISS from MNeSV (**Figure 4C**), it can be observed that the G downstream of the apical loop in bulge 2 and A and G in bulge 1 of MNeSV seem to be localized at similar positions in both YSSs. This prediction may suggest that binding of eIF4F to YSS also occurs through the eIF4E subunit as shown for the ISS, PTE and TED. This, however, has to be further studied.

## 3′-Cites are Functionally Independent Elements that Can Be Transferred Among Viruses By Recombination with Important Adaptive Consequences

As previously observed by several authors and reviewed by Simon and Miller (2013), different 3′-CITEs can be found in viruses belonging to the same family, the same genus, the same species and even the same isolate. For example, BTEs can be found in viruses belonging to different families, such as in dianthoviruses from the *Tombusviridae* family and in luteoviruses from the *Luteoviridae* family. Also, in the genus *Carmovirus* four different 3′-CITEs have been identified, TED, ISS, YSS, and TSS, and perhaps more will be discovered, as the RNA structure prediction of the 3′-end of several carmoviruses has not allowed for the identification of any of the known 3′-CITEs. Additionally, in isolates MNSV-N and PEMV2 two and three, respectively, different 3′-CITEs have been identified, belonging to different classes (Nieto et al., 2011; Gao et al., 2014; Miras et al., 2014).

The diversity of 3′-CITEs in closely related viruses suggested that these RNA elements may be interchanged between different viruses. To test this hypothesis, Nicholson et al. (2013) experimentally exchanged viral 3′-CITEs. They replaced the YSS of the tombusvirus CIRV with the ISS from the tombusvirus MNeSV and the PTE from the aureusvirus *Cucumber leaf spot virus* (CLSV) and the BTE from *Tobacco necrosis virus* (TNV-D). While maintaining the sequence complementarity between the 3′-CITE and the 5′-UTR, these chimeric viruses were able to translate and multiply efficiently, with the exception of the BTE-substitution, suggesting that 3′-CITEs are functionally interchangeable elements.

**FIGURE 5:**
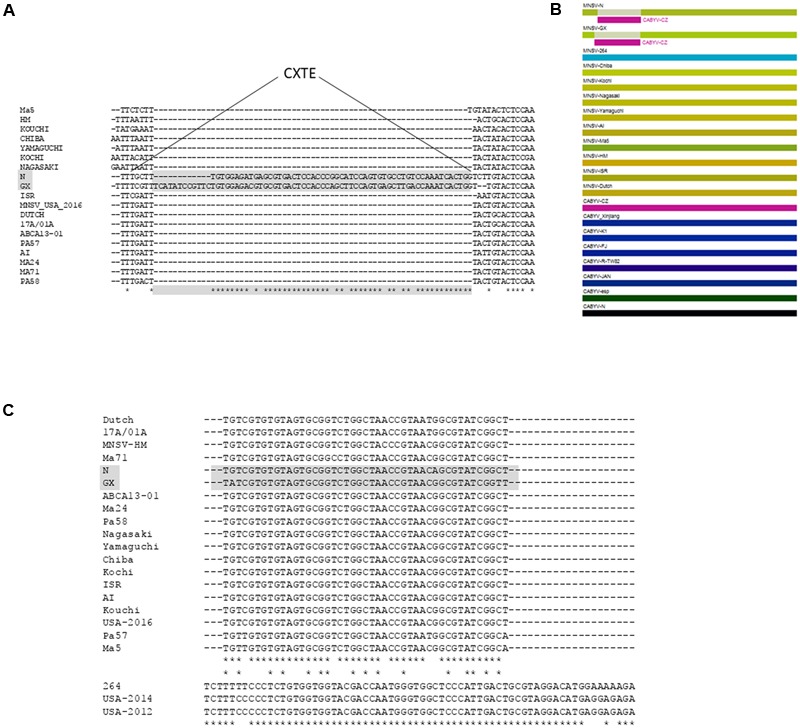
New 3′-CITEs acquired by MNSV isolates through recombination. **(A)** Sequence alignment of the 5′-end of the 3′-UTR of MNSV isolates. GenBank accession numbers of MNSV sequences included in the alignment are Ma5-AY122286, HM-GU480022.1, Chiba-AB250684, Yamaguchi-AB250687, Nagasaki-AB250686, Al-DQ339157, Dutch-NC001504, ISR (Israel)-DQ922807, Kochi-AB250685, Kouchi-AB189943, Ma24-EU589616, Ma71-EU589619, Pa58-EU589620, Pa57-EU589621, 17A/01A-M29671, ABCA13-01-KR094068, USA-2016-KY124137, N- KF060715, GX-KY264755. The inserted sequence is marked in gray. Asterisks mark conserved nucleotides of all sequences or in gray of the insertions. **(B)** Proposed recombination event generated by the RDP4 software, a computer program for characterizing recombination events in sequence alignments using several different recombination analysis methods and tests for recombination hot-spots. The sequences included in this analysis are the 3′-UTRs of MNSV [see part **(A)** of this figure] and CABYV isolates [GenBank accession numbers are CABYV-Xinjiang: EU636992; CABYV-Beijing: EU000535; CABYV esp (JF939814), CABYV-N (NC_003688), CABYV-K1 (LC082306), CABYV-FJ: GQ221223; CABYV-JAN: GQ221224; CABYV-R_TW82: JQ700306]. RDP4 colors similar sequences with similar colors. The statistical significance is very high, with a *P*-value < 0.001. **(A,B)** Of this Figure is based on our data derived from Miras et al. (2014). **(C)** Sequence alignment of the 3′-CITEs of MNSV isolates. GenBank accession numbers as above, plus MNSV-264 (AY330700), USA12 (KY124136), USA14 (KY124135), based on our data derived from Truniger et al. (2008). In the middle, the upper line with asterisks marks conserved nucleotides between sequences above; the lower asterisks line marks conservation between upper and lower sequences and asterisks line at the bottom shows nucleotides conserved between MNSV-264 and USA-2012/4.

From an evolutionary perspective, the absence of correlation between virus taxonomy and the presence of different classes of 3′-CITEs (Simon and Miller, 2013) suggests independent 3′-CITE evolutionary patterns and frequent exchange through recombination. In line with this, independent RNA recombination events appear to be responsible for the acquisition of different 3′-CITEs by MNSV field isolates. In one case, the 3′-UTR of the isolate MNSV-264, different to those from all other MNSVs, had probably been acquired by recombination from an unknown source (Nieto et al., 2011). In another case, an insertion through natural recombination of exactly a 3′-CITE was found to have occurred in another MNSV isolate, MNSV-N: here not the whole 3′-UTR, but only 55 nt were acquired by recombination of MNSV with an Asiatic CABYV isolate (**Figure 5**). This stretch of 55 nt was shown to act itself as a 3′-CITE in presence of the 5′-UTR *in cis* (Miras et al., 2014). The transferred sequence corresponds to the minimal 3′-CITE version, as its activity was lost when shortened (Miras et al., 2014). Interestingly, three new MNSV isolates have been very recently reported that could possibly result from similar recombination processes: MNSV-GX, which contains an insertion in the 5′-end of its 3′-UTR that is similar to the one from MNSV-N (**Figures 5A,B**) and MNSV-USA-2012 and 2014, that have low sequence identity with MNSV-264, although their 3′-UTRs are highly similar (**Figure 5C**). All these MNSV variants suggest that acquisition of 3′-CITEs through recombination might be frequent and/or positively selected for.

In the case of MNSV-N and -264, the recombinant viruses gained the ability to infect resistant melon (Truniger et al., 2008; Miras et al., 2014). Recessive resistance to MNSV in melon has been shown to be caused by a single amino acid change in eIF4E, resulting in loss of susceptibility to this virus while the eIF4E resistance allele is functional for cap-dependent translation (Nieto et al., 2006). While the 3′-CITE present in most MNSV isolates, Ma5TE, is eIF4E-dependent and not functional in the presence of the eIF4E resistance allele, the one from MNSV-264 and the CXTE are functional in eIF4E-silenced melons (Truniger et al., 2008; Rodríguez-Hernández et al., 2012; Miras et al., 2014). Additionally, in the case of MNSV-264, this isolate is able to infect *Nicotiana benthamiana*, a non-host for MNSV, thanks to its new 3′-CITE (Nieto et al., 2011).

## Conclusion and Prospects

Despite being considerably diverse in sequence and structure, most 3′-CITEs appear to facilitate translation through similar mechanisms including recruitment of components of the cellular translational machinery to the 3′-CITE and delivery of these through RNA–RNA interactions to the 5′-end, where the ribosome enters onto the viral RNA. BTEs have been shown to interact with the eIF4G subunit of eIF4F, while TED, ISS, and PTE 3′-CITEs interact with eIF4E. But for these 3′-CITEs for which factors involved have been identified, the order of binding and actions required for efficient cap-independent translation have still to be determined. Data obtained by high-resolution structures on how the eIFs bind the 3′-CITE are also required to understand its translation enhancer activity. Still, for several 3′-CITEs their mechanisms of action are still completely unknown.

3′-CITES are modular, functional RNA elements that can be exchanged between viruses, and their acquisition may contribute to a gain of function resulting in a selective advantage, broadening the virus host range. Interestingly, in MNSV-N, the CXTE has been acquired by interfamilial recombination with CABYV, a virus belonging to a different family (*Luteoviridae*); it is possible that this sequence also acts as a 3′-CITE in CABYV. Thus, some classes of 3′-CITEs appear in viruses of different genera or even families, although others are quite conserved within virus genera, like the YSSs that have been identified only in tombusviruses, or BTEs which have been identified in phylogenetically related viruses (Simon and Miller, 2013). This may reflect functional constraints specifically affecting 3′-CITEs, or be a consequence of the biology of each viral species. In any case, this raises questions on the spread of 3′-CITEs and their origin: 3′-CITEs as such have only been described in a few genera of plant viruses. But are they present in other plant virus genera and families, in viruses infecting other organisms, or are they even part of a conserved mechanism of translation of mRNAs of an early origin and are spread among kingdoms? The presence of 3′-CITEs in different viral genomes leads to questioning if cellular mRNAs could use similar strategies to initiate cap-independent translation, functional under stress conditions when cap-dependent translation is inhibited. Weingarten-Gabbay et al. (2016) used high-throughput approaches to identify thousands of sequences involved in cap-independent translation in both human and viral genomes. Data revealed that the human 5′- or 3′-UTRs of a set of mRNAs contained sequences involved in cap-independent translation and these were not mutually exclusive, but their mechanism remains unknown. The structural diversity of 3′-CITEs prevents high-throughput bioinformatics identification, but as 3′-CITE conformational information progresses and new search algorithms are devised, the chances of identifying 3′-CITEs in mRNAs of varied species increase.

## Author Contributions

VT wrote review. MM and MA edited and added specific information to all sections.

## Conflict of Interest Statement

The authors declare that the research was conducted in the absence of any commercial or financial relationships that could be construed as a potential conflict of interest.
